# Exploring potential targets for natural product therapy of DN: the role of SUMOylation

**DOI:** 10.3389/fphar.2024.1432724

**Published:** 2024-10-04

**Authors:** Jingjing Wang, Rui Zhang, Chenguang Wu, Lifan Wang, Peng Liu, Ping Li

**Affiliations:** ^1^ Renal Division, Heilongjiang Academy of Chinese Medicine Sciences, Harbin, China; ^2^ Shunyi Hospital, Beijing Hospital of Traditional Chinese Medicine, Beijing, China; ^3^ China-Japan Friendship Hospital, Beijing, China

**Keywords:** diabetic nephropathy, sumoylation, natural products, ginkgolic acid, ginkgolide B, resveratrol, astragaloside IV

## Abstract

Diabetic nephropathy (DN) is a common and serious micro-vascular complication of diabetes and a leading cause of end-stage renal disease globally. This disease primarily affects middle-aged and elderly individuals, especially those with a diabetes history of over 10 years and poor long-term blood glucose control. Small ubiquitin-related modifiers (SUMOs) are a group of reversible post-translational modifications of proteins that are widely expressed in eukaryotes. SUMO proteins intervene in the progression of DN by modulating various signaling cascades, such as Nrf2-mediated oxidative stress, NF-κB, TGF-β, and MAPK pathways. Recent advancements indicate that natural products regulating SUMOylation hold promise as targets for intervening in DN. In a previous article published in 2022, we reviewed the mechanisms by which SUMOylation intervenes in renal fibrosis and presented a summary of some natural products with therapeutic potential. Therefore, this paper will focus on DN. The aim of this review is to elucidate the mechanism of action of SUMOylation in DN and related natural products with therapeutic potential, thereby summarising the targets and candidate natural products for the treatment of DN through the modulation of SUMOylation, such as ginkgolic acid, ginkgolide B, resveratrol, astragaloside IV, etc., and highlighting that natural product-mediated modulation of SUMOylation is a potential therapeutic strategy for the treatment of DN as a potential therapeutic strategy.

## 1 Introduction

Diabetes represents a significant challenge to global health. This issue affects over 500 million individuals worldwide. The global prevalence of this condition is on a startling upward trajectory, with projections indicating a rise to 700 million by the year 2045 (Smith et al., 2022). Particularly, the elderly demographic faces a heightened risk of developing diabetes-related complications, including Diabetic Nephropathy (DN) (Lin et al., 2022). DN is a prevalent micro-vascular complication among individuals with diabetes and serves as a primary mechanism leading to end-stage renal disease (Shoeib et al., 2023). The pathological characteristics of DN encompass thickening of the glomerular basement membrane, mesangial matrix expansion, interstitial fibrosis in the renal tubules, and podocyte loss (Harlan et al., 2018; Wang H. et al., 2021). In individuals with diabetes, the lifetime prevalence of DN exceeds 50% (Pop-Busui et al., 2022). Factors such as hypertension (Skov et al., 2016), hyperglycemia (Wu et al., 2023), oxidative stress (Ma et al., 2021), advanced glycation end products (AGEs), and angiotensin II can induce the onset of DN by activating pathways like transforming growth factor β (TGF-β), nuclear factor kappa B (NF-κB), Nrf2-mediated oxidative stress, and mitogen-activated protein kinases (MAPK) (Hwang et al., 2019). To date, the treatment for DN has primarily focused on correcting the metabolic dysregulation and hemodynamic abnormalities in patients with diabetes. However, clinical studies have demonstrated that these therapeutic measures only partially alleviate the pathology of DN (Gross et al., 2005). Evidence suggests that standard treatments aimed at strict control of blood sugar and blood pressure are insufficient to halt the progression of DN to end stage renal disease (ESRD) (Arora and Singh, 2013). Consequently, the search for new therapeutic targets to prevent and treat DN has emerged as a critical challenge in this field.

**FIGURE 1:**
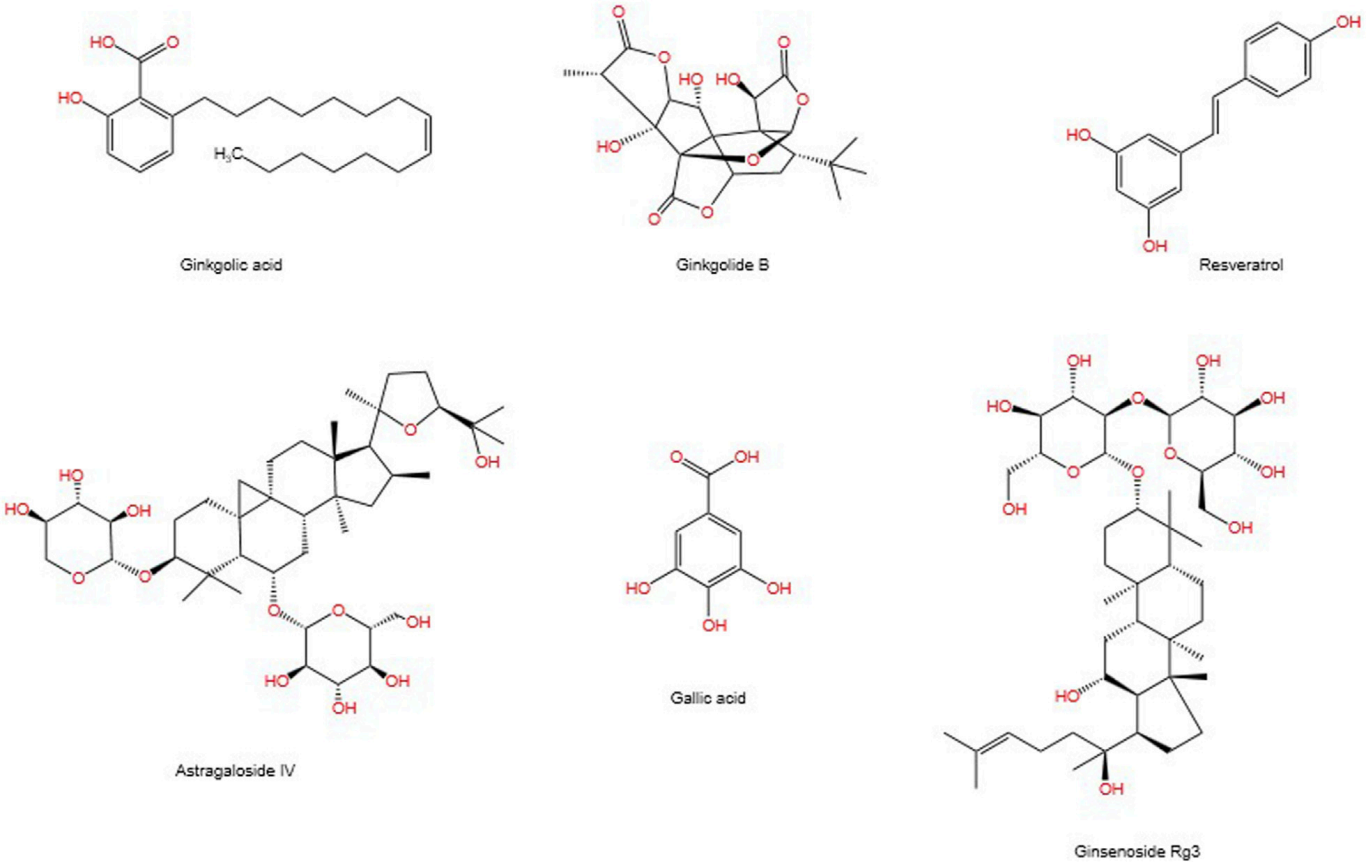
Chemical structures of selected natural compounds.

SUMOylation is a reversible post-translational modification (PTM) process (Han et al., 2018), intricately linked with various cellular processes including nucleocytoplasmic transport (Melchior et al., 2003), transcriptional regulation (Muller et al., 2004), apoptosis (Choi et al., 2017), protein stability, stress response, and cell cycle progression (Eifler and Vertegaal, 2015). It has been demonstrated that SUMO can alter and influence the function of specific metabolic enzymes in the pathway, thereby regulating the entire metabolic pathway (Gareau and Lima, 2010). Previous studies have demonstrated that SUMO is associated with a number of diseases related to metabolic disorders, including Type I diabetes (Li et al., 2005; Wang and She, 2008), Type II diabetes (Dou et al., 2022), and diabetes-mediated cardiovascular diseases (Chang and Abe, 2016). The SUMOylation E2 conjugase UBC9 was first reported to be expressed in the kidneys in 2003 (Golebiowski et al., 2003). A growing body of research suggests that SUMOylation is associated with the progression of DN through the modulation of multiple signalling pathways (Gao et al., 2014). It is also linked to the damage process of podocyte (Li et al., 2019). Although some natural products have been proven to inhibit enzymes involved in the SUMOylation process, progress in developing more selective and potent inhibitors of SUMOylation remains limited (Brackett et al., 2020). In previous publications, we have reviewed the mechanisms involved in modulating SUMOylation for the treatment of renal fibrosis, as well as candidate natural products (Liu P. et al., 2022). This article connects the mechanisms of SUMOylation with natural products, exploring the therapeutic prospects of SUMOylation in the progression of DN and the candidate natural products. Figure 1 depicts the chemical structures of the pertinent natural compounds. Table 1 lists the targets associated with SUMOylation for these compounds.

**TABLE 1:** Natural products and therapeutic targets against DN SUMOylation.

Compounds	Resource	SUMO proteins or enzymes	Therapeutic target	References
Ginkgolic acid	Ginkgo	SUMO1, SUMO2/3	SUMO1-p53	Tan et al. (2022)
Resveratrol	Vitis L., Veratrum L., Arachis, Polygonum	SUMO1 SUMO1	COX-2 β-catenin	Cheng et al. (2018), Wang et al. (2020)
Astragaloside IV	Astragalus membranaceus	SUMO1 --	HIF-1α PPARγ	Wang et al. (2021c), Xing et al. (2021)
Gallic acid	gallnut, sumac, tea leaves, and oak bark	SENP1	--	Taghvaei et al. (2022)
Ginsenoside Rg3	Panax ginseng	E3	NF-κB	Zou et al. (2018)

## 2 SUMOylation

### 2.1 SUMO proteins

Small ubiquitin-related modifiers (SUMOs) are members of the ubiquitin-like protein family and can conjugate with a wide array of proteins. Predominantly localized in the nucleus, SUMOs are crucial for various cellular processes, including cell cycle progression, genome stability, and transcription (Vertegaal, 2022). Sumoylation of proteins can influence their stability and enzymatic activity, alter their localization, or mediate novel interactions with other proteins containing SUMO-interacting motifs (SIMs) (Geiss-Friedlander and Melchior, 2007). Research has shown that the PTM of proteins by SUMOs can regulate chromatin structure and function at multiple levels, influencing gene expression and maintaining genomic integrity through various mechanisms. Both SUMO ligases and deconjugating enzymes are key factors in modulating chromatin structure. Sumoylation plays a multifaceted role in regulating chromatin architecture, gene expression, and genomic stability. Part of its function lies in the diverse downstream effects that occur when SUMO is conjugated to different proteins (Cubenas-Potts and Matunis, 2013).

SUMOs, approximately 11 kDa in size and slightly larger than ubiquitin, can bind to target proteins as single or multiple monomers or in various polymeric forms. Like ubiquitin, SUMO can undergo self-modification, such as phosphorylation and/or acetylation. The SUMO conjugation mechanism involves three types of enzymes: the activating E1 enzyme, the conjugating E2 enzyme, and the E3 ligase. In addition to covalent attachment to substrates, SUMO can interact with other proteins through SIMs. Non-covalent protein-protein interactions between sumoylated proteins containing SIMs and SUMO readers can alter the subcellular localization of the sumoylated proteins, including facilitating SUMO-mediated phase separation. SIMs in SUMO-targeted ubiquitin ligases (STUbLs) enable binding to poly-sumoylated proteins, which are subsequently ubiquitinated and degraded, forming a SUMO-mediated protein turnover pathway. Additionally, when SUMO monomers effectively compete with ubiquitin for lysine residues on target proteins, they can prevent the degradation of these proteins (Vertegaal, 2022). The specificity of the SUMO pathway is achieved through redox regulation, acetylation, phosphorylation, or other post-translational modifications of SUMOylation and deSUMOylation enzymes.

SUMO-1 typically modifies substrates as a monomer; however, SUMO-2/3 can form poly-SUMO chains. Monomeric SUMO-1 or poly-SUMO chains can interact with other proteins via SIMs, thus providing a platform to enhance protein interactions. The consequences of SUMOylation include changes in cellular localization, protein activity, or protein stability. Moreover, SUMO can facilitate protein degradation by binding ubiquitin through STUbLs (Chang and Yeh, 2020). To date, five genes encoding SUMO paralogs, known as SUMO1, SUMO2, SUMO3, SUMO4, and SUMO5, have been identified in the human genome (Wu and Huang, 2023). SUMO-1 shares only 56% identity with SUMO-2 and -3. SUMO2 and SUMO3 are 95% homologous, collectively referred to as SUMO2/3 (Kim and Baek, 2009). Mice with a knockout of the SUMO1 gene can survive, thanks to compensatory mechanisms involving SUMO2 or SUMO3 (Wang et al., 2014a; Evdokimov et al., 2008). Under different physiological and pathological conditions, the conjugation of the same substrate protein with different SUMO isoforms can lead to distinct functional outcomes. For instance, the modification of Drp1 by SUMO1 and SUMO2/3 has opposing effects on mitochondrial apoptosis regulation. SUMO1-conjugated Drp1 significantly accelerates mitochondrial fission, ultimately promoting the apoptotic process. In contrast, SUMO2/3-conjugated Drp1 effectively delays mitochondrial fission and inhibits apoptosis (Sheng et al., 2021). Similarly, in the regulation of the stability and function of the circadian rhythm protein Period2 (PER2), SUMO2 modification promotes proteasomal degradation of PER2, whereas SUMO1 conjugation inhibits its degradation and enhances its function as a transcriptional repressor (Chen et al., 2021). Additionally, the poly-SUMO2/3 modification of mutant CFTR protein associated with cystic fibrosis promotes its degradation, while SUMO1 conjugation enhances CFTR stability (Gong et al., 2019). Generally, SUMO1 modification promotes the dissolution of aberrant proteins and inhibits their aggregation, thereby protecting cells, a function particularly significant in preventing protein misfolding-related diseases. On the other hand, SUMO2/3, in coordination with STUBL and the ubiquitin-proteasome system, facilitates substrate degradation, helping cells eliminate damaged or unnecessary proteins. This mechanism is especially important in response to oxidative stress, DNA damage, and other stress conditions (Wang and Matunis, 2023). Although SUMO1, SUMO2, and SUMO3 all play important roles in cellular regulation, current studies suggest that SUMO2/3 may be more critical in the progression of DN. Unlike SUMO1, SUMO2/3 is more readily induced under conditions of cellular stress, particularly in response to oxidative stress and inflammation, which are closely associated with the high-stress environment of DN. SUMO2/3 has the ability to form poly-SUMO chains, thereby influencing a broader network of proteins. This feature gives SUMO2/3 a unique advantage in regulating complex cellular functions such as cell proliferation, differentiation, and apoptosis, making it especially relevant in the context of DN progression. SUMO4 is most similar to SUMO2/3 but contains a unique proline residue (P90) at its C-terminus, which impedes its effective maturation (Owerbach et al., 2005). Under normal culture conditions, SUMO4 is rapidly degraded; however, during cellular stress, SUMO4 can mature through stress-induced endogenous hydrolases and covalently conjugate to its substrate proteins (Wei et al., 2008). Genetic studies have linked SUMO4 to both type 1 and type 2 diabetes, suggesting that the unbound form of SUMO4 may play a role (Guo et al., 2004; Lin et al., 2007), although this association remains controversial (Podolsky et al., 2009). Nonetheless, recent research by Nisha Sinha and colleagues, through gene polymorphism analysis in diabetic patients without nephropathy (DM) and those with DN, has demonstrated that the SUMO4 c.163 G> A polymorphism is associated with an increased susceptibility to DN in North Indian patients with type 2 diabetes (Sinha et al., 2016). A recent meta-analysis has confirmed that the SUMO4 (M55V) variant is associated with both type 1 and type 2 diabetes in Asian and European populations (Li et al., 2017). The newest member of the SUMO family, SUMO5, highly homologous to SUMO1, has been shown to regulate the dynamics of PML nuclear bodies when exogenously expressed in cells (Liang et al., 2016).

### 2.2 SUMOylation and deSUMOylation process

**FIGURE 2:**
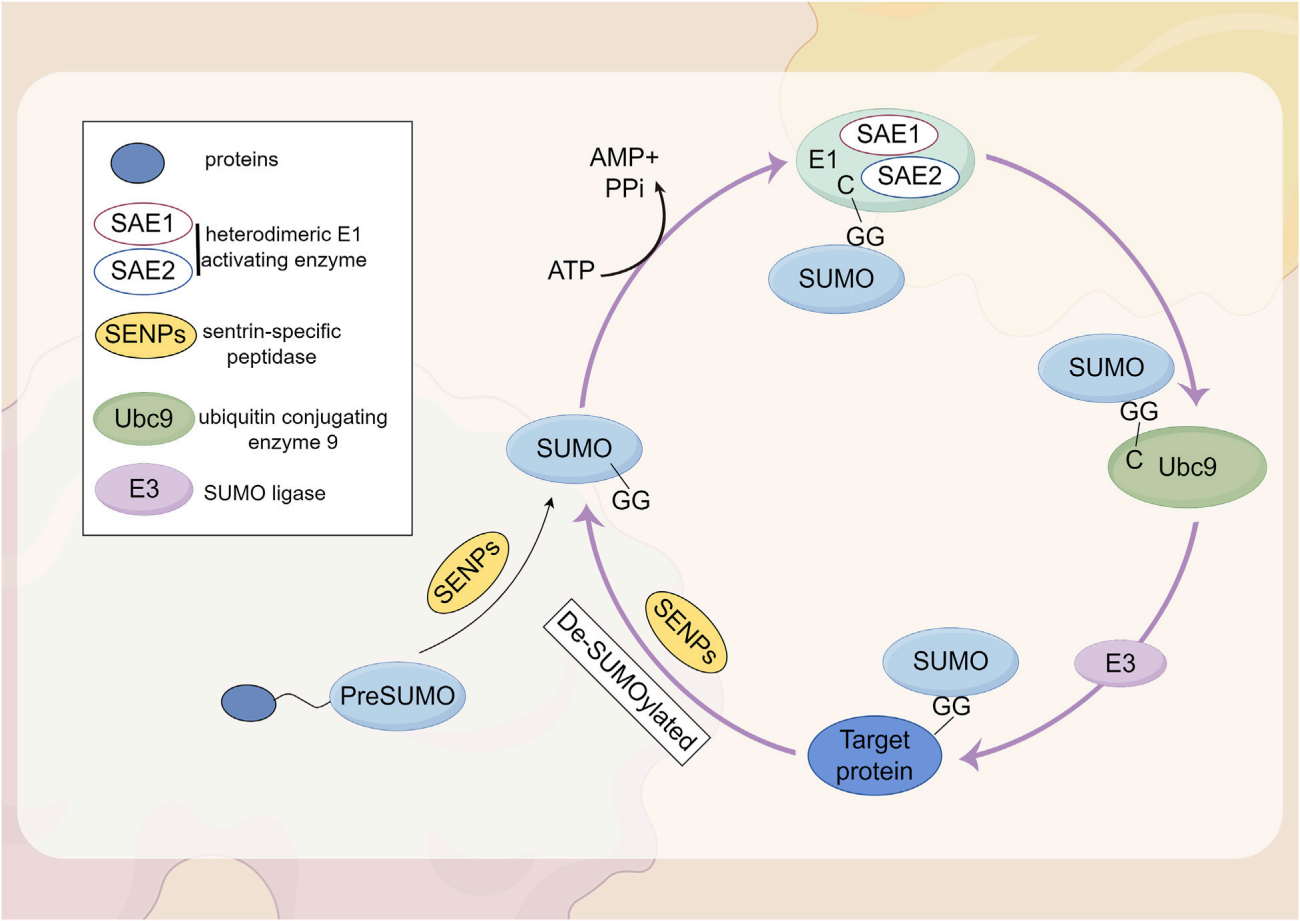
Biochemical processes by which proteins undergo SUMOylation.

SUMOylation is a reversible PTM involving the covalent attachment of small ubiquitin-like modifier proteins to substrate proteins (Tokarz and Wozniak, 2021). The attachment of SUMO1-SUMO3 to proteins, known as SUMOylation, is mediated by a mechanism that involves the dimeric SUMO activating enzyme subunits 1 and 2 (SAE1-SAE2; also known as UBA2-AOS1), the E2 conjugating enzyme UBC9 (also referred to as UBE2I), and E3 SUMO ligases, whereas deSUMOylation is regulated by the sentrin/SUMO-specific protease (SENP) family (Kunz et al., 2018; Yeh, 2009). The first SUMO proteases described were the ubiquitin-like proteases 1 and 2 (Ulp1 and Ulp2) in yeast (Li and Hochstrasser, 1999; Li and Hochstrasser, 2000). The mammalian SENP family consists of six SUMO proteases (SENP1-3 and SENP5-7), which were identified based on their sequence similarity to the Ulp family. These enzymes are all cysteine proteases and contain a catalytic triad (Cys-His-Asp) within a conserved protease domain (Jia et al., 2019). Based on sequence homology, substrate specificity, and subcellular localization, these six SENPs can be categorized into three subfamilies (Kumar and Zhang, 2015). SENP1 and SENP2 form the first subfamily. SENP1 is primarily localized in the nucleus, where it is involved in the maturation of SUMO precursors and the deconjugation of SUMOylated proteins. SENP2 is found in both the nucleus and the nuclear envelope, regulating the SUMOylation of nuclear pore components and other nuclear proteins. Both SENP1 and SENP2 exhibit broad specificity for SUMO-1, SUMO-2, and SUMO-3 and are highly homologous to the yeast Ulp1 protease. By processing the precursors of SUMO-1, SUMO-2, and SUMO-3, SENP1 and SENP2 play a multifunctional role in the SUMOylation process, which is crucial for the regulation of gene expression, DNA repair, and cell cycle progression (Kunz et al., 2018). SENP3 and SENP5 belong to the second subfamily of SUMO proteases and are also located in the nucleus. They exhibit a preference for SUMO-2 and SUMO-3 over SUMO-1, thereby regulating the function and localization of SUMOylated proteins (Yun et al., 2008; Gong and Yeh, 2006; Di Bacco et al., 2006). SENP6 and SENP7 are members of the third subfamily, typically localized in the nucleus. Their catalytic domains contain four conserved loop insertions that are absent in other SENPs, which are believed to confer their SUMO subtype specificity and preference for cleaving SUMO2/3 chains (Claessens and Vertegaal, 2024). Subsequently, the SUMO proteases deSUMOylating isopeptidase 1 (DESI1), DESI2, and USPL1 were identified, which share little sequence homology with the Ulp/SENP family (Schulz et al., 2012; Shin et al., 2012). SUMO is conjugated to lysine residues on target proteins through an isopeptide bond, catalyzed by SUMO-specific activating (E1), conjugating (E2), and ligating (E3) enzymes (Chang and Yeh, 2020). In mammalian cells, SUMOs are translated as inactive precursors and are processed into their mature forms by specific neutral proteases known as SENPs. The maturation of SUMO involves the hydrolysis of a peptide bond at the C-terminus, thereby exposing a diglycine (GG) motif. This motif subsequently forms a thioester bond with the SUMO E1 enzyme (SAE1/UBA2) in an ATP-dependent manner. Subsequently, SUMO is transferred to the catalytic cysteine residue of the SUMO E2 enzyme UBC9 through a transesterification reaction. UBC9 then conjugates SUMO to a lysine residue on the substrate, either with the assistance of a SUMO-E3 ligase or independently (Claessens and Vertegaal, 2024). The complete process of SUMOylation and deSUMOylation is illustrated in Figure 2.

## 3 Mechanisms of SUMOylation intervention in DN

**FIGURE 3:**
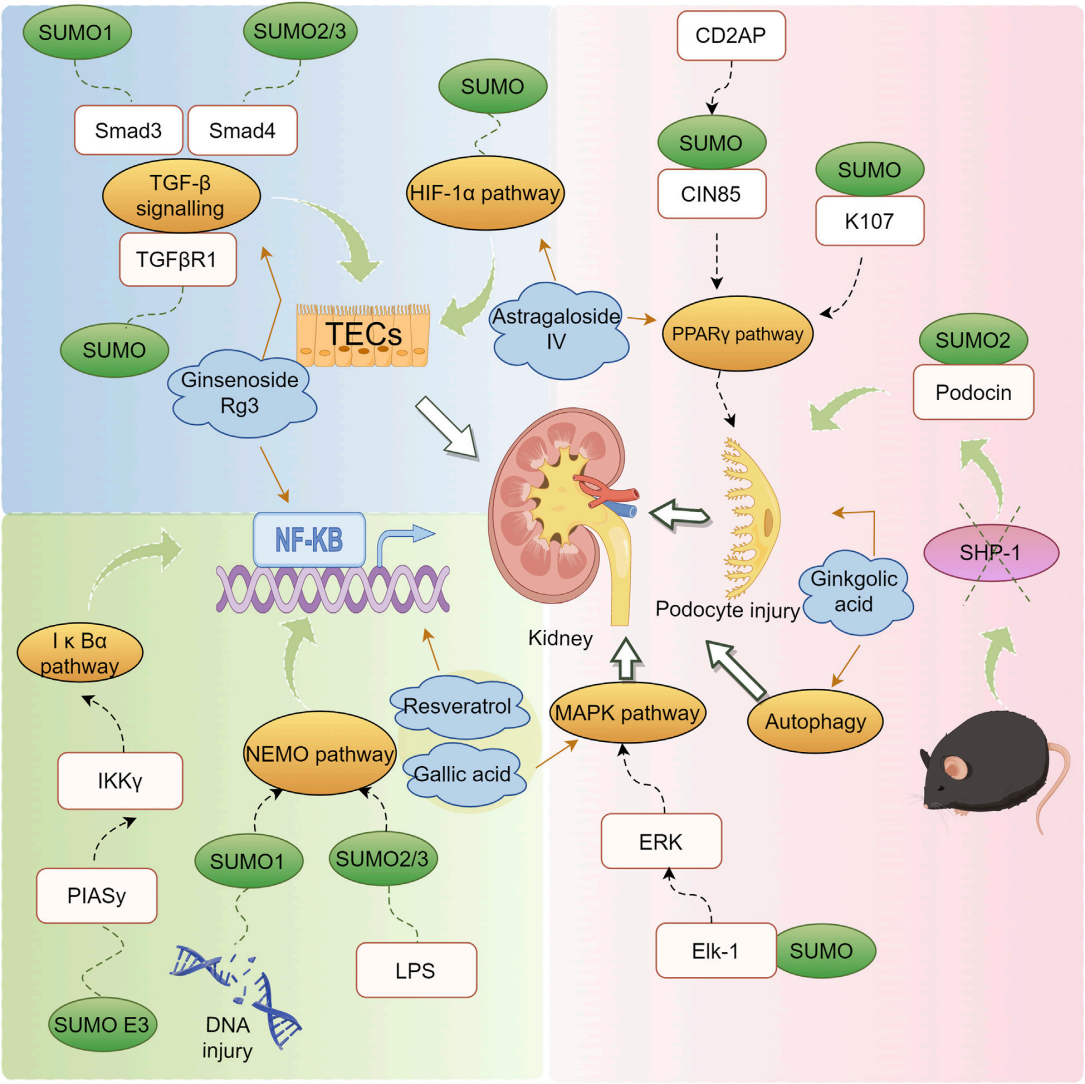
Mechanism of action of natural products in the treatment of DN through modulation of the SUMO pathway.

DN is a principal cause of End-Stage Renal Disease (ESRD) worldwide and is a major contributor to the morbidity and mortality among individuals with diabetes (Reutens and Atkins, 2011). The clinical intervention for DN primarily focuses on controlling risk factors such as hyperglycemia, dyslipidemia, hypertension, and proteinuria, aiming to alleviate symptoms and slow the progression of DN. However, these interventions have limited efficacy (Tang et al., 2021; Xiong and Zhou, 2019; Magee et al., 2017). SUMOylation has been found to be extensively involved in the pathogenesis of DN. Under normal physiological conditions, SUMOylation and deSUMOylation are maintained in dynamic balance, regulating critical cellular functions such as protein stability, transcriptional control, and DNA repair. However, in the context of DN, this balance is disrupted, leading to abnormal increases or decreases in SUMOylation levels. Modification of the NR5A2 protein, a key mediator in the transcriptional regulation of the calreticulin gene, by SUMO exacerbates renal fibrosis (Arvaniti et al., 2016). Furthermore, SUMO may play a protective role against the apoptosis of podocyte (Yang et al., 2019). These findings have been summarized in detail (Figure 3; Table 2).

**TABLE 2:** The SUMOylation of transcription factors and key mediators in DN.

*In vivo/In vitro*	Model	SUMO proteins or enzymes	Target genes	References
*In vivo*	Inducible beta cell-specific Ubc9- mice	UBC9	NRF2	He et al. (2018)
*In vivo/In vitro*	Human islets and C57BL/6 mice	SENP1	NADPH and GSH	Ferdaoussi et al. (2015)
*In vitro*	islets and β-cells, INS-1 832/13 cells	SENP1	synaptotagmin VII	Dai et al. (2011)
*In vitro*	MIN6 cell	SUMO-1	GLP-1R	Rajan et al. (2012)
*In vivo/In vitro*	Senp2knockout mice, C3H10T1/2 cell line	SENP2	CREB	Liang et al. (2019)
*In vivo/In vitro*	C57BL/6 mice, ob/ob mice, db/db mice, INS1 cells and the human islets	SENP2	Cyclin D1 and Mafa	Jung et al. (2016)
*In vivo/In vitro*	ancreatic β cell-specific Senp2 knockout mice, NIT-1 cells	SENP2	DRP1	Nan et al. (2022)
*In vivo*	inducible adipocyte-specific Ubc9 knockout mice	Ubc9	ERp44	Xie et al. (2023)
*In vivo/In vitro*	C57BL/6 Senp3^+/−^ mice and Senp3flox mice, RAW264.7, HEK293T, and TLR4 HEK293 cells	SENP3	MKK7 kinase	Lao et al. (2018)
*In vivo/In vitro*	C57BL/6 SENP3^fl/fl^ mice and Lyz2‐Cre SENP3 cKO mice, RAW264.7 cells	SENP3	JNK	Chen et al. (2020)
*In vivo*	C57BL/6 mice, Senp3^flox/flox^ control mice, and Senp3^flox/flox^; Lyz2-Cre mice	SENP3	YAP1	Jiang et al. (2023)
*In vivo/In vitro*	C57BL/6 mice, HEK293T, MEF and RAW264.7 cells	SENP6	NEMO	Liu et al. (2013a)
*In vitro*	HepG2 cells, SMMC7721 cells	SUMO1	p65	Liu et al. (2016)
*In vivo/In vitro*	IB3-1 cell lines, A549 and 16HBE cell lines, CF mice	SUMO1	TG2	Luciani et al. (2009)
*In vitro*	Rat glomerular mesangial cells	E3 Ligase PIASy	NF-κB	Huang et al. (2017)
*In vivo*	Rat GMCs (HBZY-1)	SUMO1 and SUMO2/3	IκBα	Huang et al. (2013)
*In vitro*	HEK293, COS7 and 3T3 cells	SUMO4	IκBα	Guo et al. (2004)
*In vivo/In vitro*	UUO.Smad3-null mice, renal tubular epithelial cells, bone marrow monocytes	Smad3	TGF-β	Sato et al. (2003)
*In vivo*	Smad3^ex8/ex8^ mice and streptozotocin-inducedCL57/Bl6J mice	Smad3	TGF-β	Fujimoto et al. (2003)
*In vivo*	Smad3-KO mouse	Smad3	TGF-β	Wang et al. (2007)
*In vivo/In vitro*	Smad4 KO mice, mouse model of unilateral ureteral obstruction, Smad4 KO macrophages and fibroblasts	Smad4	Smad3 and Smad7	Meng et al. (2012)
*In vitro*	Rat glomerular mesangial cells (HBZY-1)	SUMO2/3	Smad4	Zhou et al. (2014)
*In vitro*	Hep3B cell line, HaCaT cell line and 293T cell line	Smad3	Smad3	Imoto et al. (2008)
*In vitro*	NIH 3T3 cells	Smad4	TGF-β	Long et al. (2004)
*In vivo*	SENP2 deficiency mice	SENP2	ERK5 and p53	Heo et al. (2013)
*In vivo/In vitro*	C57/Bl6 mice, mouse podocytes and human embryonic kidney 293 cells	SUMO-1 and SUMO-2/3	nephrin	Tossidou et al. (2014)
*In vitro*	conditionally immortalized mouse CD2AP^+/+^and CD2AP^−/−^podocytes	SUMO-1 and SUMO-2/3	CIN85	Tossidou et al. (2012)
*In vivo/In vitro*	C57BL/6 mice and the MPC5 cell line	SENP6	Notch1, EDN1	Guo et al. (2023)
*In vivo*	SHP-1-deficient mice	SUMO2	SHP-1	Lizotte et al. (2023)
*In vitro*	Immortalized mouse podocytes	SENP1	p53	Wang et al. (2014b)
*In vitro*	GEnC cell line	SENP1	HIF-1α	Wang et al. (2015a)
*In vitro*	SRA01/04 cells	E3	HIF-1α	Han et al. (2015)

### 3.1 SUMOylation interferes with insulin secretion

**FIGURE 4:**
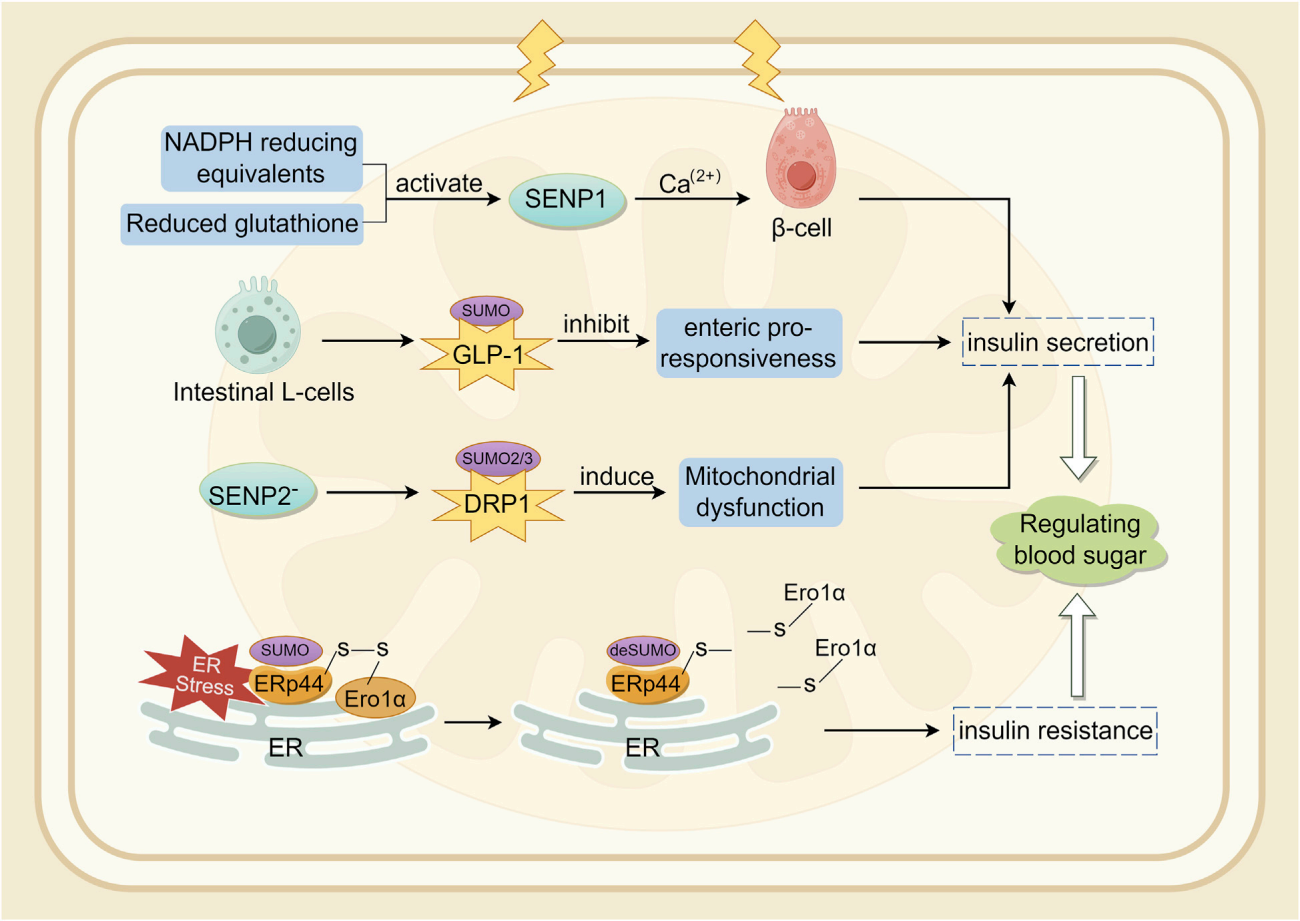
Involvement of SUMOylation in insulin secretion.

The SUMO pathway is associated with the production and secretion of insulin (Kamynina and Stover, 2017). In a diabetic mouse model established through UBC9 deficiency, β-cells exhibited impaired antioxidant capacity, and Ubc9 deficiency resulted in reduced NRF2 activity and decreased expression of its downstream antioxidant genes, leading to Reactive Oxygen Species (ROS) accumulation and oxidative stress (He et al., 2018). SENP1 plays a crucial role in insulin secretion in type 2 diabetes (Ferdaoussi et al., 2015). SENP1 is essential for glucose-dependent insulin secretion. SUMOylation responds to intracellular Ca^2+^ elevation by directly and reversibly inhibiting β-cell exocytosis, thereby acutely regulating insulin secretion (Dai et al., 2011; Manning et al., 2012). NADPH equivalents and reduced glutathione activate SENP1 function, thereby increasing insulin secretion (Ferdaoussi et al., 2015). Conversely, over-expression of SENP1 reduces insulin secretion and impairs the handling of Ca^2+^-induced cell death (Hajmrle et al., 2014). Glucagon-like peptide-1 (GLP-1) is an incretin hormone secreted by intestinal L-cells, crucial for postprandial insulin secretion (Drucker et al., 1987). In mouse pancreatic β-cell lines and primary mouse β-cells, cAMP produced by GLP-1R stimulation was shown to be downregulated by SUMO. Therefore, SUMO modification of GLP-1R may be a contributing factor to the diminished incretin response (Rajan et al., 2012). Oxidative stress-mediated inactivation of SENP1 may be a critical step in impaired insulin secretion in type 2 diabetes (Shoeib et al., 2023; Attie, 2015). Studies indicate that SENP2 is involved in adipocyte differentiation (Liang et al., 2019) and adipogenesis (Chung et al., 2010), adipose tissue thermogenesis (Liu et al., 2021), and insulin sensitivity (Zheng et al., 2018). Expression of SENP2 in insulin-secreting cells is triggered by chronic high glucose stimulation. *In vitro*, the increase in SENP2 levels under high glucose conditions was more pronounced in the cytoplasm than in the nucleus (Jung et al., 2016). Experiments have shown that SENP2 regulates mitochondrial function and insulin secretion in pancreatic β-cells, with the regulation of DRP1 sumoylation-mediated phosphorylation being a potential mechanism (Nan et al., 2022). Endoplasmic reticulum protein 44 (ERp44) is closely related to glucose and lipid metabolism (Nyirimigabo et al., 2019). Interfering with ERp44 SUMOylation enhances ERp44 degradation and impairs ER retention by Ero1α, thereby ameliorating ER stress and maintaining adipose tissue homeostasis. Therefore, modulating ERp44 SUMOylation in adipocyte may be a viable strategy to combat obesity and insulin resistance (Xie et al., 2023). A synopsis of the preceding discussion is provided in Figure 4.

### 3.2 Improvement of the inflammatory response

Inflammation is closely related to the development and progression of DN (Deepa and Venkatraman, 2013). A significant body of research indicates that the activation of inflammatory signaling and the infiltration of inflammatory cells are crucial for the development of DN (Galkina and Ley, 2006). Increasing evidence suggests that SENPs play roles in modulating macrophage function and inflammation. For instance, Lao et al. (2018) reported that SENP3 enhances lipopolysaccharide (LPS) induced TLR4 signal transduction via the deSUMOylation of MKK7, and myeloid-specific deletion of SENP3 can alleviate LPS-induced endotoxic shock and acute lung injury (Chen et al., 2020). During high-fat diet (HFD)-induced obesity in mice, SENP3 expression in adipose tissue macrophages (ATMs) is significantly increased, while myeloid-specific SENP3 deficiency protects mice from HFD-induced obesity and systemic inflammation. This protective effect is partly mediated by alterations in YAP1 sumoylation. The YAP1 signaling pathway plays a role in regulating macrophage function and inflammation (Zhou et al., 2019; Liu M. et al., 2020). In the context of HFD-induced obesity in mice, SENP3 regulates the deSUMOylation of YAP1, while the absence of SENP3 can abolish the upregulation of YAP1 induced by IL-1β. Myeloid-specific deletion of SENP3 attenuates macrophage infiltration in adipose tissue and reduces serum inflammatory factor levels during diet and age-related obesity (Jiang et al., 2023).

#### 3.2.1 NF-κB signaling

As a pivotal coordinator of inflammatory responses, NF-κB controls the expression of various inflammatory cytokines associated with the pathogenesis of DN (Perez-Morales et al., 2019; Wu et al., 2016). Some components of the NF-κB pathway can be modified by the SUMOylation process (Mabb and Miyamoto, 2007). NEMO can be considered a potential target for downregulating NF-κB activity, as NF-κB activation requires SUMO modification of NEMO before its accumulation in the nucleus (Hamdoun and Efferth, 2017). SUMO1 is conjugated by Ubc9 to K21 and K22 lysines of IκBα, preventing its ubiquitination and degradation, and further inhibiting the activation of NF-κB. Specifically, the NEMO kinase, a part of the IKK complex essential for NF-κB activation, is modified by SUMO1 in response to DNA damage, while exposure to lipopolysaccharide induces the modification of NEMO by SUMO2/3. This modification prevents the deubiquitinase CYLD from binding to NEMO, thereby enhancing the activation of the NF-κB essential modifier (IKK) (Liu X. et al., 2013). P100 can also activate the NF-κB pathway upon SUMOylation, with NEMO SUMOylation involving SUMO-1, mainly regulating the activation of NF-κB in genotoxic responses (Liu et al., 2016). PIAS proteins enhance the SUMOylation of tissue TG2, keeping TG2 stable and avoiding ubiquitin-mediated degradation, thereby sustaining oxidative stress and chronic inflammation in cells (Luciani et al., 2009). It has been elucidated that elevated glucose upregulates PIASy expression and that the SUMO E3 ligase PIASy mediates high-glucose-induced NF-κB inflammatory signalling, suggesting that PIASy is a potential therapeutic target for DN (Huang et al., 2017). Studies have found that high glucose stimulation enhances the expression of SUMO1 and SUMO2/3 in a dose- and time-dependent mannerand significantly reduces SUMOylation of IκBα, thereby activating NF-κB signalling (Huang et al., 2013). SUMO 4 binds to IκBα and negatively regulates NF-κB transcriptional activity (Guo et al., 2004).

#### 3.2.2 TGF-β signaling

TGF-β is a multifunctional cytokine recognized, with Smad4 identified as a central channel in the TGF-β signaling mechanism (Wang L. et al., 2021). In the unilateral ureteral obstruction (UUO) mouse model, the absence of Smad3 significantly reduces renal fibrosis (Sato et al., 2003). Regarding DN, in streptozotocin (STZ)-induced DN models, mice lacking Smad3 were found to avoid renal fibrosis, including glomerular basement membrane (GBM) thickening and excessive extracellular matrix (ECM) production (Fujimoto et al., 2003), although the suppression of albuminuria was consistently observed (Wang et al., 2007). Studies have shown that Smad4 knockdown enhances pro-inflammatory NF-κB signalling while inactivating fibrotic Smad3 signalling (Meng et al., 2012). High glucose significantly increases the expression of SUMO1 and SUMO2/3 and promotes the conjugation of SUMO2/3 to Smad4 (Zhou et al., 2014). In addition to Smad4, Smad3 also plays an important role in coordinating TGF-β-mediated signalling processes. Studies have verified that PIASy, acting as a SUMOylation E3 ligase, intervenes in TGF-β signaling by binding to and SUMOylating Smad3, thus serving as a regulatory checkpoint (Derynck et al., 1998; Imoto et al., 2008). PIASy regulates TGF-β/Smad3-mediated signaling by stimulating the SUMOylation and nuclear export of Smad3. SUMOylation also inhibits the transcriptional activity of Smad4 (Gao et al., 2014; Long et al., 2004).

#### 3.2.3 MAPK signaling

The Mitogen-Activated Protein Kinase (MAPK) pathway is a crucial conduit for transmitting external signals to internal cellular responses and is recognized as a prototypical pro-inflammatory signaling pathway. The over-activation of the MAPK pathway has been closely linked to numerous inflammatory diseases. Indeed, several MAPK inhibitors have undergone preclinical evaluation in a wide range of disease models, showing significant therapeutic potential (Yong et al., 2009). This pathway primarily participates in various cell functions by regulating the phosphorylation of its substrates, a process closely associated with SUMOylation (Guo et al., 2007). In conditions of chronic hyperglycemia, the MAPK pathway is activated, leading to localized inflammatory responses (Sakai et al., 2005). Recent research has revealed that the inhibition of the transcription factor Elk-1 due to SUMOylation can be counteracted by activating the ERK pathway (Besnard et al., 2011). Moreover, the SUMOylation of key proteins such as p53 and ERK5 has profound effects, influencing the apoptosis of endothelial cells under disturbed blood flow and regulating inflammatory processes (Heo et al., 2013).

### 3.3 The SUMOylation of podocyte apoptosis pathway

Clinical and experimental studies have shown that podocytopenia leads to proteinuria and/or glomerulosclerosis. And apoptosis is the main reason for the decrease in podocyte numbers (Asanuma, 2015; Nagata, 2016). Podocyte injury is an early critical event in the progression of DN, in which autophagy is an important factor (Xu et al., 2020; Deretic, 2021). This degradation process involves pathways such as mTOR, AMPK, and PI3K, playing a crucial role in inhibiting inflammation and kidney damage, thereby slowing the progression of DN (Zhang Z. et al., 2022).

Podocyte injury, including ultra-structural changes and reduced expression of slit diaphragm components like nephrin, podocin, and CD2AP, underlies many glomerular diseases. Nephrin is a SUMO-1 and SUMO-2/3 SUMOylated target protein and the presence of SUMOylation increases nephrin stability (Tossidou et al., 2014). CD2AP affects the post-translational structure of CIN85 in podocyte, enhancing its modification by SUMO-1, -2, and -3. Converting lysine 598 to arginine eliminates this modification and enhances the interaction between CIN85 and nephrin, indicating a newly discovered regulatory function of CD2AP (Tossidou et al., 2012). SENP6 deficiency enhances podocyte loss induced by high glucose (HG) through activation of the Notch1 signaling. Simultaneously, the lack of SENP6 promotes the production of EDN1 by regulating the transcription of EDN1 in podocyte, thereby exacerbating HG-induced glomerular endothelial cell injury and dysfunction. This suggests that the protective role of SENP6 may be related to its deSUMOylation function (Guo et al., 2023).

SHP-1 is a cell membrane protein tyrosine phosphatase expressed primarily in haematopoietic and epithelial cells. In the kidney, increased expression of SHP-1 in mouse and human podocytes exposed to HG leads to decreased insulin and nephrin action and podocyte dysfunction (Drapeau et al., 2013; Lizotte et al., 2016). Research has demonstrated that SHP-1 can decrease the PTM of podocin by SUMO2. In renal tissues of diabetic mice and patients, a reduction in SUMO2 and an increase in SUMO1 are observed. Upon deletion of SHP-1 in podocyte, the SUMOylation of structural proteins is restored, thereby maintaining their integrity and mitigating the progression of DN. Thus, targeting SHP-1 could represent a strategic approach to prevent podocyte injury caused by diabetes (Lizotte et al., 2023).

In kidney disease, p53 has been identified as a key factor in regulating podocyte apoptosis (Wada et al., 2005). Data indicate that SENP1 expression was low in normal podocyte and significantly increases in podocyte undergoing apoptosis and oxidative stress induced by puromycin aminonucleoside (PAN). The absence of SENP1 results in the accumulation of SUMOylated p53, which directly mediates increased apoptosis in podocytes treated with PAN (Wang et al., 2014b).

Renal tubular hypoxia is a major driving factor in DN proximal tubular pathology (Miyata and de Strihou, 2010). Hypoxia-inducible factor (HIF)-1 plays a crucial role under hypoxic conditions. There is increasing evidence that HIF-1α expression is elevated in renal proximal tubule cells under hyperglycaemic conditions (Bessho et al., 2019). Elevated levels of ROS may lead to renal cell damage, resulting in renal dysfunction (Liang et al., 2021). It was shown that HIF-1α attenuates high glucose-mediated associated renal tubular cell injury by promoting Parkin/PINK1-mediated mitosis (Yu et al., 2021). Hypoxia might promote the stability and activation of HIF-1α by increasing the expression of SENP1 in podocyte, thereby inducing survival and angiogenesis in Glomerular Endothelial Cells to combat hypoxia. The deSUMOylation of HIF-1α signaling protects glomerular endothelial cells from hypoxic injury (Wang L. et al., 2015). Over-expression of SUMO or SUMO E3 ligase can enhance the stability and transcriptional activity of HIF-1α in Human Lymphatic Endothelial Cells (Han et al., 2015).

Although the current study reveals beneficial effects of SENP1, it is important to consider the insights from other studies. For instance, Meinecke *et al.* demonstrated that elevated levels of SUMO acetylation in promyelocytic leukaemia proteins result in resistance to Fas-induced apoptosis (Meinecke et al., 2007). In a study conducted by Li et al. (2008) it was demonstrated that the deSUMOylation of HIPK1 by SENP1 in TNF-treated human umbilical vein endothelial cells resulted in the enhancement of ASK1-dependent apoptosis. These contradictory results are intriguing and, aside from the different types of stimuli and signaling pathways studied, might also relate to the different cell types investigated. Therefore, the relationship between SUMOylation and apoptosis warrants further exploration.

## 4 Natural products that combat DN by regulating SUMOylation

In the clinical treatment of DN, pharmacological interventions are being explored alongside natural products. Traditional drug therapies focus primarily on blood glucose control, blood pressure management, and inhibition of the renin-angiotensin-aldosterone system (RAAS). Antidiabetic medications like metformin and SGLT2 inhibitors have shown some degree of nephroprotective effects. Additionally, ACE inhibitors (ACEIs) and angiotensin II receptor blockers (ARBs) are effective in reducing proteinuria and slowing the progression of renal dysfunction (Hu et al., 2023a). However, these synthetic drugs are often accompanied by side effects, including ketoacidosis, weight gain, polyuria, liver damage, renal impairment, and cardiovascular complications (Roborel et al., 2020; Blahova et al., 2021; Kushwaha et al., 2020a). This has prompted further exploration of phytochemicals as alternative therapeutic approaches (Kushwaha et al., 2020b). Phytochemicals are considered potential natural modulators due to their ability to interact with multiple targets, slow disease progression, and their relatively low toxicity (Khatoon et al., 2022). Among these, natural compounds like polyphenols, flavonoids, and saponins possess antioxidant, anti-inflammatory, and antifibrotic properties, offering protective effects on the kidneys (Hu et al., 2023b). Research suggests that certain natural compounds and herbal extracts may exert therapeutic effects on DN through mechanisms such as SUMOylation, highlighting their potential as a treatment option for DN.

Ginkgo biloba is a traditional herbal medicine with thousands of years of clinical use history and stands as one of the most widely used botanical treatment products globally (Howes and Houghton, 2012). It is primarily utilized for treating conditions such as cough, asthma, and enuresis (Nash and Shah, 2015). Previously, researchers found that ginkgo biloba alleviated the worsening of proteinuria in patients with type 2 diabetes, suggesting that ginkgo biloba is a promising option for early protection of the kidneys in patients with DN (Howes and Houghton, 2012). Modern pharmacological research indicates that Ginkgo biloba possesses anti-oxidative, anti-inflammatory (Mohanta et al., 2014), neuroprotective (Zuo et al., 2017), and anti-platelet aggregation effects (Koch, 2005). Animal studies have confirmed Ginkgo biloba’s preventive and therapeutic effects on DN (Avula et al., 2015; Qiu et al., 2015; Tang et al., 2009). Ginkgolic acid (GA), an alkylphenol constituent found in Ginkgo biloba leaves and fruits (Isah, 2015), significantly reduces and reverses the modulation induced by oxidized low-density lipoprotein, indicating its anti-inflammatory action through the NF-κB pathway (Zhang and Yan, 2019). GA is a SUMO chemical inhibitor that binds directly to the E1 enzyme and inhibits the formation of the E1-SUMO intermediate, thereby effectively inhibiting the SUMO chemical pathway (Fukuda et al., 2009). Research has demonstrated that this compound can downregulate the expression of SUMO1 and SUMO2/3 in macrophages, an effect consistently replicated in both experimental and clinical models (Liu X. et al., 2022). The application of GA to inhibit SUMOylation or suppress SUMO1 expression can regulate the levels of SUMO1 and its conjugation with p53, promoting autophagy while inhibiting cell proliferation. These results reveal the ability of GA to control the multifaceted regulation of complex cellular processes (Tan et al., 2022). Beyond its cell regulatory functions, SUMOylation is also essential for the exchange on slit diaphragms, a critical component of the renal unit in kidney architecture. Treatment with the SUMOylation inhibitor GA reduces the expression of nephrin on podocyte membranes, highlighting the importance of SUMOylation in renal homeostasis and its potential as a therapeutic intervention target (Tossidou et al., 2014).

Research indicates that polyphenols hold promise for treating various chronic diseases, including diabetes and its complications, with minimal toxic effects observed *in vitro* and in animal models (Kabir et al., 2021). Resveratrol (RES) is a naturally occurring non-flavonoid polyphenolic compound derived mainly from natural plants such as grapes, cranberries, lingonberries and certain herbs (Nanjan and Betz, 2014). The pharmacological actions of RSV have been studied, including its anti-oxidative, anti-inflammatory, immunomodulatory, hepatoprotective, anticancer, and anti-atherosclerotic effects (Pandey and Rizvi, 2009; Prasad, 2012; Bo et al., 2013; Carrizzo et al., 2013; Carter et al., 2014; Wang Y. et al., 2015; Peiyuan et al., 2017). Studies have shown that RSV treatment has a protective effect on DN, with RSV treatment in animals with DN alleviating hyperglycemia, hyperlipidemia, and improving kidney structure integrity and renal function (Den Hartogh and Tsiani, 2019). Resveratrol effectively inhibits carcinogen-induced expression of cyclooxygenase-2 (COX-2). This process requires the phosphorylation of extracellular signal-regulated kinases 1 and 2 (ERK1/2) and the translocation of the enzyme to the nucleus by SUMO-1. COX-2 then accumulates in the nucleus, forming a complex with SUMO-1, which binds to phosphorylated p53, potentially influencing the carcinogenic process (Cheng et al., 2018). Furthermore, resveratrol can mediate the downregulation of SUMO1, inhibit the expression of Wnt5a, deactivate β-catenin, and prevent their entry into the cell nucleus. This molecular cascade attenuates the Wnt/β-catenin signaling pathway, thereby producing an anti-inflammatory effect (Wang et al., 2020). Experimental evidence suggests that resveratrol may be an effective agent for the prevention and inflammatory response of renal mesangial cells induced by LPS and the expression of fibronectin through the SphK1/S1P2/NF-κB signaling pathway, highlighting resveratrol’s potential role in combating renal fibrosis in addition to its anti-hyperglycemic properties (Gong et al., 2020).

Astragalus membranaceus, known as Huangqi, is the dried root of Astragalus and a commonly used herb in traditional Chinese medicine. Increasing research demonstrates its beneficial effects on various diseases through activating immune mechanisms, alleviating oxidative stress, and reducing inflammation (Cho and Leung, 2007; Ko et al., 2005; Qin et al., 2012). Astragaloside IV (AS-IV), a saponin extracted from Astragalus membranaceus Bunge, is one of the main pharmacologically active components of Astragalus (Fu et al., 2014). Recent studies report that AS-IV possesses extensive pharmacological activities both *in vivo* and *in vitro*, including anti-oxidative stress, anti-inflammatory, anti-diabetic, and renal protective effects (Zhou et al., 2017; Chen et al., 2018). Further research utilizing advanced Western blotting techniques has found that the sharp decline and delayed recovery of SUMO1 activity are responsible for the initial increase and subsequent decrease in HIF-1α concentration. Enhanced expression of SUMO1 stabilizes HIF-1α within the nucleus, effectively reducing the extent of vascular anomalies following hypoxia. Astragaloside IV (AS-IV) triggers sustained production of SUMO1 in vascular endothelial cells, consolidating the HIF-1α/VEGF axis and thus promoting angiogenesis under hypoxic conditions (Wang B. et al., 2021). Additionally, AS-IV seems to alleviate podocyte apoptosis by activating the PPARγ-Klotho-FoxO1 signaling pathway, offering therapeutic potential for DN (Xing et al., 2021). Peroxisome proliferator-activated receptor γ (PPARγ), a target of thiazolidinediones used to treat patients with type 2 diabetes mellitus (T2DM), plays a crucial role in enhancing insulin sensitivity, promoting adipogenesis and exerting anti-inflammatory effects (Sauer, 2015; Stechschulte et al., 2016; Rao et al., 2015). In cell-based studies, the SUMOylation of the N-terminal K107 on PPARγ has been shown to inhibit its transcriptional activity, suggesting that targeting this modification could be a strategic approach in drug therapy. This strategy aims to separate the beneficial insulin-sensitizing properties of PPARγ from its adverse effects on body weight (Katafuchi et al., 2018). Extensive research indicates that PTM of PPARγ regulate its transcriptional activity, with SUMOylation being a crucial negative regulatory mechanism (Ohshima et al., 2004). Studies have demonstrated that excessive SUMOylation of PPARγ, similar to the effects of HFD, induces endothelial insulin resistance (IR) and dysfunction by negatively regulating the eNOS-NO pathway. More importantly, it was discovered that excessive SUMOylation of PPARγ triggers an endogenous SUMOylation cascade, exacerbating endothelial IR and dysfunction (Yuan et al., 2019).

Gallic acid (GA) is a bio-active compound found, known for its antioxidant, anti-allergic, anti-cancer, anti-inflammatory, and anti-diabetic properties (Chen et al., 2009; D’Souza et al., 2014; Huang et al., 2012; Hidalgo et al., 2012; Ho et al., 2013; Habtemariam, 1997; Nabavi et al., 2013; Punithavathi et al., 2011; Liu K. Y. et al., 2013). In human diets, the primary sources of this compound are non-glycosylated esters of gallic acid (Wianowska and Olszowy-Tomczyk, 2023). Gallic acid is a potent phenolic substance involved in crucial deSUMOylation processes through SENP1, particularly targeting GATA2, NEMO, Pin1, SMAD4, and HIF-1α, thus having significant impacts on various diseases (Taghvaei et al., 2022). Studies have shown that gallic acid effectively inhibits the renal activation of p38 MAPK and nuclear NF-κB, ultimately alleviating renal dysfunction in diabetic rats induced by a combination of HFD and STZ through p38 MAPK-mediated pathways (Ahad et al., 2015). Toxicological investigations indicate that gallic acid exhibits minimal toxicity or adverse reactions across a range of animal experiments and clinical studies (Bai et al., 2021).

Ginsenosides, the primary active components extracted from the roots, stems, leaves, or fruits of ginseng, are extensively cultivated in Korea and Northeast China (Sui et al., 2023). Ginseng has been reported to have anti-diabetic properties due to its ability to induce insulin secretion, stimulate glucose uptake, inhibit intestinal glucose absorption, and reduce glycogen breakdown (Chen et al., 2012; Yuan et al., 2012). Ginsenoside Rg3 has been shown to effectively protect against hyperglycemia, obesity, and diabetes by preventing the death of pancreatic β-cell (Kim et al., 2016; Kim et al., 2009). In a supplementary study, an eight-week treatment with 20(R)-Rg3 not only lowered fasting blood glucose levels and harmful levels of advanced glycation end-products but also beneficially regulated insulin modulation, lipid profiles, oxidative markers, and overall kidney health. This was achieved by affecting the MAPK and NF-κB signaling cascades in DN mice, highlighting 20(R)-Rg3 as a promising therapeutic agent (Li et al., 2021). Additionally, the well-known ginsenoside Rg3 has been identified as up-regulating the phosphorylated RanBP2 (an E3 SUMO-protein ligase), thereby exerting inhibitory effects on the NF-κB signaling pathway and offering new therapeutic avenues for cancer treatment (Zou et al., 2018). This inhibitory effect may be related to the regulation of nucleocytoplasmic transport. The phosphorylation of RanBP2 might affect its interaction with Nup153, leading to the aggregation of these nucleoporins. This aggregation could prevent IκB from entering the nucleus, thereby prolonging its binding to the p65 subunit and inhibiting NF-κB activity (Liu Y. et al., 2020).

In addition to natural products, TAK-981 and ML-792 are small-molecule SUMO inhibitors with significant selectivity. Research has shown that the SUMOylation inhibitors TAK-981 and ML-792 stimulate cytotoxic NK cells, M1 macrophages, and CTLs, while also preventing the exhaustion of CD8^+^ T cells, thereby enhancing antitumor activity (Wang et al., 2024). The SUMO E1 inhibitor TAK-981 is currently undergoing clinical trials for cancer treatment (Langston et al., 2021). The discovery of TAK-981 opens up possibilities for novel immunotherapies, offering new opportunities for the treatment of diseases such as cancer and sepsis (Zhang H. et al., 2022; Youssef et al., 2023).

Recent research indicates that the activation of the Notch, Wnt, and Hedgehog (Hh) signaling pathways plays a crucial role in the regeneration of damaged organs. However, excessive stimulation of these pathways can lead to fibrosis development. Therefore, reducing the activity of Notch, Wnt, or Hh signaling may represent a new therapeutic strategy for DN (Edeling et al., 2016). The Wnt pathway, particularly the canonical Wnt pathway, is considered a key regulator in the progression of DN. In DN, the TGF-β and Wnt signaling pathways mutually activate and regulate each other, promoting tissue fibrosis and damage repair, which exacerbates disease progression (Wang H. et al., 2021). At the same time, glucocorticoid receptors (GR) in endothelial cells play a key role in regulating renal fibrosis by inhibiting the canonical Wnt signaling pathway (Srivastava et al., 2021). Research on DN has shown that the Notch signaling pathway significantly affects podocytes and tubular epithelial cells (Yang and Liu, 2022; Ma et al., 2022). Additionally, fibroblast growth factor receptor 1 (FGFR1) signaling is involved in regulating kidney damage. Studies suggest that FGFR1 promotes the fibrotic response of renal tubular epithelial cells in the context of hypertension and elevated angiotensin II, indicating that FGFR1 is a potential target for preserving kidney function and integrity (Xu et al., 2022). Sirtuin 3 (SIRT3), a major mitochondrial deacetylase, plays a crucial role in the production and detoxification of reactive ROS. Experimental results show that regulating the SIRT3 pathway can improve diabetes-induced kidney damage. Specific activation of SIRT3 reduces oxidative stress induced by diabetes, protecting podocytes and glomeruli from damage (Locatelli et al., 2020). These signaling pathways and their interactions offer potential targets for future therapeutic strategies in treating DN.

## 5 Conclusion

To conclude, the present investigation provides an integrated review of SUMOylation’s involvement in DN, alongside the medicinal actions of natural products that influence transcription factors and pivotal signaling molecules. Given the intricate nature of DN onset, which is characterized by an interplay of diverse factors and a spectrum of molecular dynamics, and is indicative of both systemic and renal-specific pathological conditions, efficacious therapeutic strategies are markedly lacking. The role of SUMOylation in DN is complex, as its effects depend on specific target proteins and pathological conditions. The application of SUMOylation regulation in DN requires further research and validation. Current studies indicate that SUMOylation levels are often elevated in DN, particularly in response to high glucose and oxidative stress. This process offers some benefits by enhancing the activity of antioxidant proteins, which temporarily protect the kidneys from further oxidative damage. However, excessive SUMOylation can also drive the activation of profibrotic and pro-inflammatory signaling pathways, such as TGF-β and NF-κB, exacerbating kidney fibrosis and chronic inflammation. Therefore, future research may need to focus on balancing SUMOylation regulation through precise therapeutic targeting. This approach would aim to prevent the excessive accumulation of SUMO modifications while preserving the necessary cellular stress responses.

Natural products are widespread and diverse in nature and may provide additional directions and options for the treatment of DN. The burgeoning research into natural products capable of modulating SUMOylation is poised to elucidate the role of SUMOylation in the progression of diabetic kidney ailments, spotlighting compounds like ginkgolic acid, ginkgolide B, astragaloside, resveratrol, gallic acid, and ginsenoside Rg3. It is important to note that the difference in specificity between natural products and modern synthetic inhibitors is significant, directly influencing their efficacy and safety in research and therapeutic applications. While the natural products mentioned in this paper can affect SUMOylation, they often exert their effects through multiple pathways that are not necessarily related to SUMO modification. In contrast, compounds like TAK981 are designed with high specificity, targeting the SUMOylation pathway by inhibiting the SUMO E1 enzyme, thereby blocking the SUMOylation process. This high specificity allows TAK981 to more precisely regulate SUMOylation. However, natural products, due to their broad availability and multi-target characteristics, can simultaneously influence multiple biological pathways, which is particularly advantageous for complex diseases. Moreover, they tend to have lower side effects and reduced risk of resistance, making them a crucial component in disease treatment. While investigations into natural products modulating SUMOylation are nascent, the pursuit of viable SUMOylation-based treatments is a considerable hurdle, necessitating extensive and detailed foundational studies.

Future research could focus on developing natural product-based drugs targeting SUMOylation pathways, with the aim of regulating specific SUMOylation processes to slow or prevent the progression of DN. Additionally, SUMOylation modulators could be combined with other DN treatments, such as SGLT2 inhibitors and RAAS inhibitors, to create multi-target therapeutic strategies. This approach could act on multiple pathological mechanisms simultaneously, improving treatment efficacy while reducing the side effects associated with monotherapies. In summary, regulating SUMOylation may open new avenues for DN treatment, especially by targeting various pathological processes to achieve more personalized and precise therapeutic interventions.
